# Evaluation of glycemic variability in chronic liver disease patients with type 2 diabetes mellitus using continuous glucose monitoring

**DOI:** 10.1371/journal.pone.0195028

**Published:** 2018-04-03

**Authors:** Fumi Honda, Akira Hiramatsu, Hideyuki Hyogo, Hiroshi Aikata, Kana Daijo, Yuji Teraoka, Yuki Inagaki, Kei Morio, Tomoki Kobayashi, Takashi Nakahara, Yuko Nagaoki, Tomokazu Kawaoka, Masayasu Yoneda, Masataka Tsuge, Michio Imamura, Yoshiiku Kawakami, Hidenori Ochi, Kazuaki Chayama

**Affiliations:** 1 Department of Gastroenterology and Metabolism, Applied Life Science, Institute of Biomedical and Health Sciences, Hiroshima University, Hiroshima, Japan; 2 Department of Gastroenterology and Hepatology, JA Hiroshima General Hospital, Hiroshima, Japan; 3 Department of Molecular and Internal Medicine, Graduate School of Biomedical Sciences, Hiroshima University, Hiroshima, Japan; Universita degli Studi Magna Graecia di Catanzaro Scuola di Medicina e Chirurgia, ITALY

## Abstract

**Background and aims:**

The feature of blood glucose dynamics in patients with chronic liver disease (CLD) is marked blood glucose fluctuations. However, the detail of blood glucose dynamics is not well known. The aim of the present study was to evaluate glycemic fluctuations by continuous glucose monitoring (CGM).

**Materials and methods:**

A total of 105 CLD patients with type 2 diabetes mellitus (T2DM) were enrolled in this study. Various parameters of glycemic variability were evaluated. The association of these parameters with liver functional reserve was examined. The parameters were also evaluated according to glycated hemoglobin A1c (HbA1c) levels.

**Results and discussion:**

Data of all patients showed that mean blood glucose (MBG) levels and the difference between highest and lowest blood glucose (ΔBG) increased significantly with worsening of liver functional reserve (*P* < 0.001 and *P* = 0.005, respectively). Although many of the cases were being treated for diabetes, postprandial hyperglycemia was seen in 92% of patients. Nocturnal hypoglycemia was seen in 22% of patients. In non-anemic patients with HbA1c levels of < 7.0%, the percentage of patients with mean amplitude of glycemic excursion (MAGE) of ≥ 77.4 mg/dL and that of MBG levels of > 145 mg/dL were higher in liver cirrhosis (LC) patients than in chronic hepatitis (CH) patients. In them, homeostasis model assessment for insulin resistance (HOMA-IR) of > 2.5 and LC were significantly associated with the increase in MAGE. LC was also significantly associated with increased MBG levels.

**Conclusion:**

The CGM systems were useful in finding hidden abnormalities of blood glucose fluctuations in CLD patients with T2DM, especially in non-anemic CLD patients with HbA1c levels of < 7.0%.

## Introduction

A large cohort study showed that diabetes was an independent risk factor for chronic liver disease (CLD) and hepatocellular carcinoma (HCC) [1]. On the other hand, CLD is one of the major causes of death in diabetic patients [2–3]. These results point to a close relationship between CLD and diabetes. The liver plays a central role in glucose metabolism. Almost all patients with cirrhosis are insulin-resistant, 60% to 80% are glucose intolerant, and about 20% develop diabetes [4]. It is important to maintain a good glycemic control status because patients with CLD and inadequate blood glucose control have poor prognosis [5]. Insulin resistance and hyperinsulinemia are metabolic features of patients with CLD. Furthermore, postprandial hyperglycemia is seen frequently in patients with CLD. On the other hand, the metabolic profiles of patients with liver cirrhosis (LC) after an overnight fast resemble those found in normal humans after 2–3 days of starvation. This phenomenon reflects the fact that hepatic glycogen store is decreased in patients with LC [6]. For these reasons, nocturnal hypoglycemia sometimes occurs in patients with LC.

The main feature of blood glucose dynamics in patients with CLD is marked blood glucose fluctuations, such as postprandial hyperglycemia and nocturnal hypoglycemia. However, the detail of blood glucose dynamics is not well known. Glycated hemoglobin A1c (HbA1c) is the gold standard for monitoring blood glucose control in diabetes. However, HbA1c does not properly represent the glycemic control status in patients with LC due to the short lifespan of erythrocytes caused by hypersplenism [7–9]. HbA1c has been shown to be apparently lower in relation to hyperglycemia in patients with CLD. On the other hand, although glycated albumin (GA) is not influenced by disorders of hemoglobin metabolism, it is affected by disorders of albumin metabolism. GA level is apparently higher in relation to hyperglycemia in CLD patients due to the prolonged half-life of serum albumin caused by reduced capacity for albumin synthesis [10–11]. Moreover, diabetic patients with similar HbA1c levels may differ in terms of glucose stability. In other words, since HbA1c is an integrated measure of overall glucose exposure, similar HbA1c levels can be generated by different glucose profiles [12]. Thus, it is difficult to monitor glycemic control status accurately in patients with CLD, because none of the known markers precisely reflects that status.

Frequent blood testing, such as self-monitoring of blood glucose (SMBG) provides diabetics with accurate and discrete blood glucose levels. However, SMBG does not provide trend information on glucose, nor reflects glycemic fluctuations. Nocturnal hypoglycemia is never captured by SMBG. On the other hand, continuous glucose monitoring (CGM) systems can provide information about glucose levels every few minutes, allowing patients to view a graph of glucose levels. Furthermore, the CGM systems provide maximum information about fluctuations in blood glucose that various parameters of glycemic variability [mean blood glucose (MBG), the difference between highest and lowest blood glucose (ΔBG), and mean amplitude of glycemic excursion (MAGE)] by continuous analysis of interstitial glucose level throughout the day, and the device is currently used to educate patients with diabetes. In Japan, the device was covered by Medicare by the Ministry of Health, Labour and Welfare in 2009.

It is known that blood glucose fluctuations are hidden in patients with CLD. Moreover, postprandial hyperglycemia and nocturnal hypoglycemia are features of blood glucose kinetics of CLD patients. The CGM systems can help identify nocturnal hypoglycemia and prevent it from being unwanted postprandial hyperglycemia with minimal invasiveness. The results of CGM may contribute to more appropriate therapeutic strategy for blood glucose control in CLD patients with type 2 diabetes mellitus (T2DM). The aim of this study was to analyze blood glucose fluctuations using the CGM in CLD patients with T2DM.

## Materials and methods

### Patients

The study protocol was approved by the Human Ethics Review Committee of Hiroshima University and a signed consent form was obtained from each subject. It complies with the Treaty of Helsinki. Between September 2013 and August 2015, 105 CLD patients with T2DM were enrolled in this retrospective study. The study subjects were patients with CLD diagnosed with T2DM according to Japan Diabetes Society guidelines [13]. T2DM was diagnosed based on the presence of one of the following criteria: (i) fasting plasma glucose level of ≥ 126 mg/dL; (ii) 2-h plasma glucose level of ≥ 200 mg/dL after 75-g oral glucose tolerance test (OGTT); or (iii) casual plasma glucose level of ≥ 200 mg/dL. According to the current revision, in addition to the above listed plasma glucose levels, HbA1c has been given a more prominent position as one of the diagnostic criteria. That is, (iv) HbA1c level of ≥ 6.5% is currently considered to indicate T2DM. All patients underwent CGM during hospitalization. Diet, including calories, was set for each individual patient during hospitalization. The selected median calorie per day was 1600 kcal (range, 1200–2000 kcal). The calorie intake and treatment contents were fixed during CGM.

### Diagnosis of chronic hepatitis and liver cirrhosis

Patients were divided clinically into chronic hepatitis (CH) group (n = 51) and liver cirrhosis (LC) group (n = 54). LC was determined radiologically or by histopathological examination of liver biopsy material. Patients who could not be diagnosed radiologically or by histopathological examination, an APRI (AST to platelet ratio index) score of > 1 was used to indicate LC. The APRI score was calculated using Wai’s formula: [(AST/upper limit of normal)/platelet count (expressed as platelets × 10^9^/l) × 100]. The reported sensitivity, specificity, and positive and negative predictive values of APRI score > 1 for LC are 89%, 75%, 38%, and 98%, respectively [14].

### Measurement of CGM

Subcutaneous interstitial glucose levels were monitored on an ambulatory basis over 72 consecutive hours by using the CGM systems (iPro2, Medtronic, Minneapolis, MN). All patients received detailed information about the benefits, examination procedure, and possible complications of the CGM. The sensor was inserted into the subcutaneous tissue of the abdomen and removed after 96 hours, with a daily record containing 288 logging continuous sensor values. In all patients, the CGM was calibrated at least 4 times per day with a SMBG device (Medisafe-Mini, Terumo, Japan). According to the operating guidelines, the CGM was installed to monitor glucose levels in the interstitial fluid. The following parameters were computed from the recording; MBG, ΔBG, MAGE, the standard deviation of blood glucose (SDBG), area under the curve of blood glucose above 140 mg/dl, (AUCgluc ≥ 140) and area under the curve of blood glucose below 70 mg/dl (AUCgluc < 70). MAGE quantified major swings in blood glucose but exclude minor fluctuations when assessing intra-day glycemic variability. MAGE represented the arithmetic mean difference between consecutive blood glucose peaks and nadirs when differences were > 1 standard deviation of the mean glucose value in the same 24 hours period [15–16]. It is reported that the mean value of MAGE of early screening-diagnosed T2DM patients based on OGTT was 77.4 mg/dL [17]. In the present study, 200 mg/dL, 70 mg/dL, and 77.4 mg/dL were defined as the cutoff values for maximum blood glucose, lowest blood glucose, and MAGE, respectively. As for MBG and ΔBG, the mean values of the subgroup were defined as the cutoff values.

### Comparison of glycemic parameters obtained from CGM

The level of HbA1c is commonly used as reliable index of glycemic control in diabetic patients [18–19]. HbA1c levels of < 7.0% is currently considered the treatment target with respect to diabetic complications [20]. However, in patients with CLD, the level of HbA1c shows lower values relative to the degree of glycemia because of anemia caused by hypersplenism-associated shorter erythrocyte lifespan. Thus, in anemic patients with CLD, the level of HbA1c is not reliable. Therefore, we divided the patients into three groups; CLD patients with HbA1c levels of ≥ 7.0% (n = 64), non-anemic CLD patients with HbA1c levels of < 7.0% (n = 29) and anemic CLD patients with HbA1c levels of < 7.0% (n = 12). In this study, we defined anemia as hemoglobin (Hb) levels of ≤ 12 g/l in men and ≤ 10 g/l in women. Because the number of anemic CLD patients with HbA1c levels of < 7.0% was few, we investigated the former two groups. The patients with HbA1c levels of ≥ 7.0% consisted of 33 CH and 31 LC patients, and the non-anemic patients with HbA1c levels of < 7.0% consisted of 14 CH and 15 LC patients. Blood glucose fluctuations, such as maximum blood glucose, lowest blood glucose, MBG, ΔBG, MAGE, SDBG, AUCgluc ≥ 140 and AUCgluc < 70 were compared between CH patients and LC patients.

### Statistical analysis

All statistical analyses were performed using SPSS for Windows 20.0 (SPSS Inc, Chicago, IL). Results are presented as frequencies and percentages for categorical variables and median (range) for continuous variables. Differences between two groups were assessed by using the chi-square and unpaired *t* tests. To ascertain the independent contribution of MAGE and mean glucose level, multivariate regression analysis was used. To assess the trend of MBG, ΔBG, MAGE, SDBG, AUCgluc ≥ 140 and AUCgluc < 70 in the sample, each parameter was compared across the three groups divided by liver functional reserve using a Jonckheere-Terpstra test. *P* value of < 0.05 was considered statistically significant.

## Results

### Patient characteristics and laboratory data

Table 1 shows the characteristics of the patients [62 men and 43 women, median age: 68 years (range, 24–84)]. There were 51 CH patients and 54 LC patients. According to the Child-Pugh classification, 31 patients were classified as class A, 18 as class B and 5 as class C. Glucose-lowering agents were already prescribed in 87 patients, and those were dipeptidyl peptidase-4 inhibitors (DPP-4I, n = 59), biguanides (BG, n = 29), sulfonylureas (SU, n = 28), α-glucosidase inhibitors (α-GI, n = 28), insulin therapy (n = 27), thiazolidinediones (n = 5), rapid-acting insulin secretagogues (n = 2) and sodium glucose cotransporter 2 inhibitors (SGLT2I, n = 1), with some patients taking more than one type of these agents. There were not significant difference blood glucose fluctuations between these glucose-lowering agents. Age, etiology, sex, BMI, and the rates of lifestyle-related diseases (hypertension, dyslipidemia and hyperuricemia) were not significantly different between CH group and LC group. The levels of AST (*P* = 0.001) and ALT (*P* = 0.049), the rates of HCC (*P* = 0.004) and APRI (*P* < 0.001) were significantly higher in LC group than in CH group. Hb levels, platelet count, and albumin levels were significantly higher in CH group than in LC group (*P* < 0.001). On the other hand, HbA1c, fasting plasma glucose (FPG), fasting immunoreactive insulin (FIRI) levels, and homeostasis model assessment for insulin resistance (HOMA-IR) were similar in the two groups. HOMA-IR, a method used to quantify insulin resistance, was calculated as follows: HOMA-IR index = FPG (mg/dL) × FIRI (mU/L) / 405. The rate of use of glucose-lowering agents except for biguanides were similar in the two groups. This is because biguanides is contraindicated in patients with severe hepatic impairment.

**Table 1:** Characteristics of patients.

	ALL patients				Patients with HbA1c ≥7.0%			Non-anemic patients with HbA1c <7.0%		
	All	CH group	LC group	*P* value	CH group	LC group	*P* value	CH group	LC group	*P* value
**Number of patients**	105	51	54		33	31		14	15	
**Age (years)**	68 (24–84)	66 (34–81)	68.5 (24–84)	0.099	66 (34–80)	69 (56–84)	0.005	68 (59–81)	68 (24–79)	0.279
**Etiology**										
**HBV/HCV**	9/30	3/9	6/21		1/5	3/9		2/4	1/10	
**Alcoholic**	25	10	15		6	9		1	4	
**NASH**	24	19	5		15	4		3	1	
**AIH**	2	0	2		0	1		0	0	
**Hemochromatosis**	1	0	1		0	1		0	0	
**Cryptogenic**	16	10	6		6	4		4	2	
**Sex (M/F)**	62/43	29/22	33/21	0.658	20/13	19/12	0.955	6/8	7/8	0.837
**Child Pugh classification (A/B/C)**			31/18/5			21/9/1			7/6/2	
**BMI (kg/m**^**2**^**)**	25.2 (15.4–67.0)	25.2 (19.2–67.0)	24.3 (15.3–48.5)	0.249	26.7 (20.0–67.0)	25.1 (15.4–36.2)	0.126	24.7 (19.2–34.8)	25.2 (17.1–48.5)	0.593
**Hb (g/dl)**	12.8 (7.5–17.1)	13.7 (8.6–17.1)	11.9 (7.5–16.8)	<0.001	13.5 (9.5–16.8)	12.8 (8.2–15.7)	0.006	14.1 (11.5–17.1)	12.2 (10.5–16.8)	0.012
**HbA1c (%)**	7.3 (4.6–16.9)	7.3 (5.6–16.9)	7.3 (4.6–13.4)	0.178	7.8 (7.0–16.9)	8.1 (7.1–13.4)	0.669	6.6 (5.6–6.9)	6.2 (4.6–6.9)	0.035
**FPG (mg/dl)**	131 (68–298)	129 (86–267)	142 (68–298)	0.679	133 (86–267)	151 (93–298)	0.789	117.5 (96–156)	106 (68–194)	0.74
**Insulin (μIU/ml)**	13.3 (1.9–59.1)	12.4 (1.9–47.5)	14.5 (2.7–59.1)	0.616	11.8 (1.9–34.3)	14.2 (2.7–59.1)	0.129	15.5 (4.7–47.5)	18.3 (3.5–44.5)	0.584
**HOMA-IR**	4.37 (0.6–30.4)	3.95 (0.60–30.4)	4.55 (1.06–27.1)	0.603	3.95 (0.60–18.2)	4.75 (1.1–27.1)	0.188	4.08 (1.33–30.4)	4.52 (1.1–10.66)	0.441
**AST (IU/L)**	34.5 (7–172)	30 (7–82)	47 (14–172)	0.001	29 (16–82)	44 (17–159)	0.017	29.5 (7–60)	50 (14–172)	0.002
**ALT (IU/L)**	35 (5–158)	33 (9–96)	39 (5–158)	0.049	33 (9–96)	39 (10–158)	0.236	24.5 (9–70)	41 (5–138)	0.026
**Albumin (g/dl)**	3.8 (2.3–5.3)	4.2 (2.9–5.3)	3.5 (2.3–4.4)	<0.001	4.2 (2.9–4.9)	3.8 (2.4–4.4)	0.001	4.5 (3.2–5.3)	3.4 (2.3–4.4)	<0.001
**Platelet count (10**^**3**^**/μl)**	143 (28–688)	184 (85–367)	101 (28–688)	<0.001	197 (85–367)	123 (34–688)	0.005	143 (103–252)	106 (28–243)	0.006
**HCC (yes/no)**	46/59	15/36	31/23	0.004	5/28	15/16	0.004	5/9	11/4	0.042
**APRI**	0.8 (0.11–10.5)	0.54 (0.15–1.01)	1.31 (0.11–10.5)	<0.001	0.43 (0.19–1.01)	1.13 (0.11–5.79)	<0.001	0.67 (0.15–0.94)	1.31 (0.2–10.5)	<0.001
**Hypertension (yes/no)**	60/45	29/22	31/23	0.955	19/14	23/8	0.162	8/6	7/8	0.573
**Hyperlipidemia (yes/no)**	29/76	18/33	11/43	0.087	13/20	7/24	0.147	4/10	2/13	0.291
**Hyperuricemia (yes/no)**	22/83	7/44	15/39	0.077	5/28	8/23	0.29	2/12	4/11	0.361
**Medications for diabetes (yes/no)**	87/18	44/7	43/11	0.367	32/1	25/6	0.043	10/4	10/5	0.55

Data are median (range) values or number of patients.

CH: chronic hepatitis, LC: liver cirrhosis, NASH: non-alcoholic steatohepatitis, AIH: autoimmune hepatitis, BMI: body mass index, HOMA-IR: homeostasis model assessment-insulin resistance, AST: aspartate aminotransferase, ALT: alanine aminotransferase, HCC: hepatocellular carcinoma, APRI: asparate aminotransferase to platelet ratio index

### CGM data according to liver functional reserve

**Fig 1:**
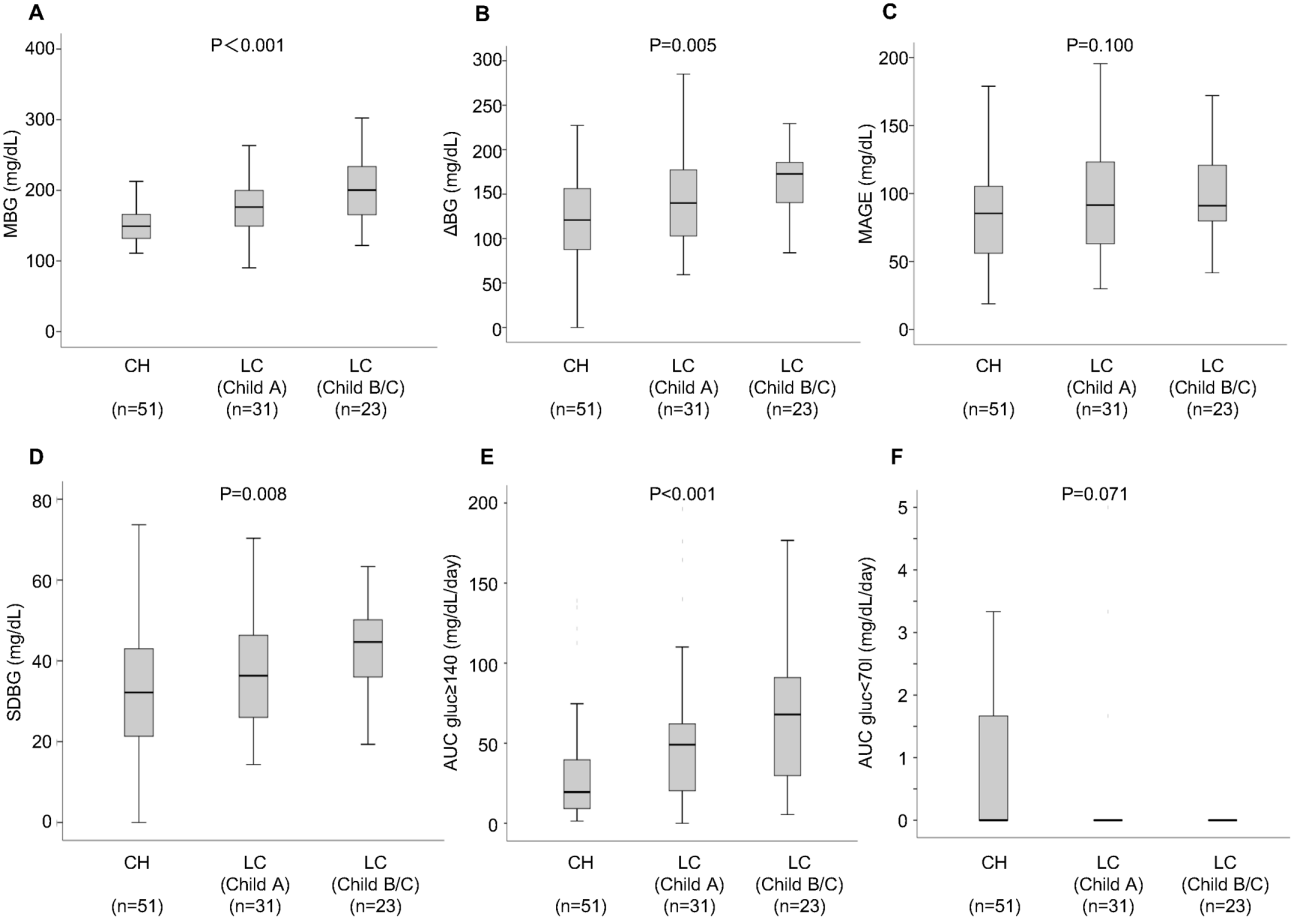
Comparison of CGM parameters in three groups according to liver functional reserve (CH group, Child A group and Child B and C group). (A) Mean blood glucose (MBG), (B) delta change in blood glucose (ΔBG), (C) mean amplitude of glycemic excursion (MAGE), (D) standard deviation of blood glucose (SDBG), (E) area under the curve of blood glucose above 140 mg/dl, (AUCgluc ≥ 140) and (F) area under the curve of blood glucose below 70 mg/dl (AUCgluc < 70) according to the severity of background liver disease in patients with chronic hepatitis (CH), Child-Pugh grade A and Child-Pugh B and C. In these box-and-whisker plots, lines within the boxes represent median values; the upper and lower lines of the boxes represent the 25th and 75th percentiles, respectively; and the upper and lower bars outside the boxes represent the 90th and 10th percentiles, respectively.

Subjects were also divided into three groups according to liver functional reserve (CH group, Child A group and Child B and C group). FPG levels and HOMA-IR did not show any significant difference between the three groups. On the other hand, HbA1c levels were significantly lower in Child B and C group than in CH group (*P* = 0.039). However, the levels of MBG, ΔBG, MAGE, SDBG and AUCgluc ≥ 140 increased steadily from CH group to Child B and C group in a stepwise manner (Fig 1). The tendency was significant for MBG (*P* < 0.001), ΔBG (*P* = 0.005), SDBG (*P* = 0.008) and AUCgluc ≥ 140 (*P* < 0.001). There was no significant difference in AUCgluc < 70 due to differences in hepatic functional reserve. The proportion of patients taking biguanide was significantly higher in CH group than in the other groups (p = 0.009). Therefore, we further performed multivariate ordinal logistic regression analysis adjusting for biguanide. Among factors which was significantly associated with hepatic functional reserve in univariate analysis, MBG, ΔBG and AUCgluc ≥ 140 were still significant after adjusting for biguanide (P < 0.001, P = 0.033, and P = 0.003, respectively), whereas SDBG was not (P = 0.115).

### Comparison of CGM parameters between CH group and LC group in patients with HbA1c levels of ≥ 7.0%

Next, we compared CGM glycemic parameters between CH group and LC group in those patients with HbA1c levels of ≥ 7.0%. Table 1 summarizes the patients’ baseline characteristics and laboratory data. This subgroup consisted of 33 CH patients and 31 LC patients. Age (*P* = 0.005), AST (*P* = 0.017), rate of HCC (*P* = 0.004) and APRI (*P* < 0.001) were significantly higher in LC group than in CH group. On the other hand, Hb levels (*P* = 0.006), platelet count (*P* = 0.005), albumin levels (*P* = 0.001) and the rate of use of glucose-lowering agents (*P* = 0.043) were significantly lower in LC group than in CH group. However, HbA1c, FPG, FIRI and HOMA-IR were not significantly different.

**Fig 2:**
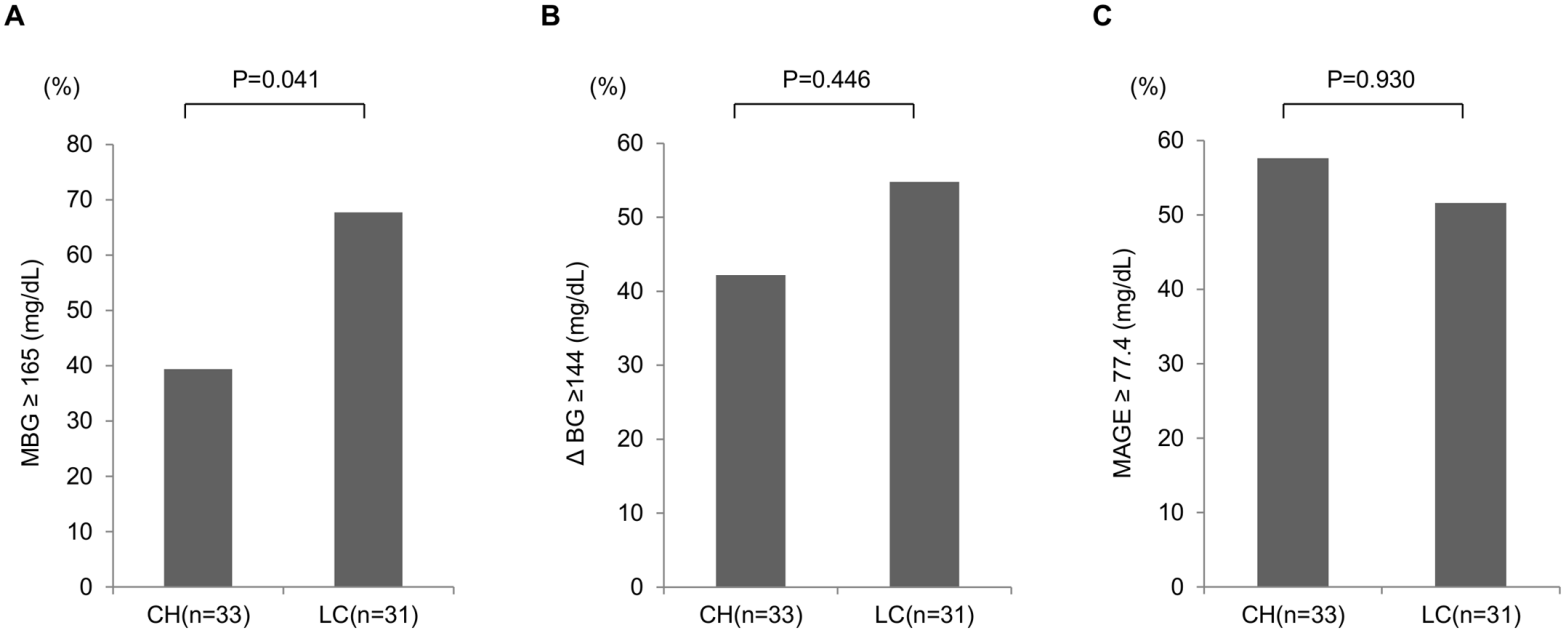
Comparison of CGM parameters between CH group and LC group among patients with HbA1c levels of ≥ 7.0%. (A) MBG, (B) ΔBG, and (C) MAGE. The population of patients whose values were more than or equal to the cutoff values were compared between CH patients and LC patients. MBG: mean blood glucose, ΔBG: the difference between the highest blood glucose and the lowest blood glucose, MAGE: mean amplitude of glycemic excursion, CH: chronic hepatitis, LC: liver cirrhosis.

We compared various parameters derived from the CGM between CH group and LC group (Fig 2). ΔBG and MAGE were not different between CH group and LC group. On the other hand, the percentage of LC patients with MBG levels of ≥165 mg/dL was significantly higher than that of CH patients (*P* = 0.041).

Next, univariate and multivariate analyses were performed to identify factors that contributed to the increase in mean glucose level in HbA1c levels of ≥ 7.0% patients (Table 2). Multivariate analysis identified LC as the only factor that was independently associated with the increase in mean glucose level (odds ratio = 2.908, 95% confidence interval = 1.031–8.204, *P* = 0.044).

**Table 2:** Results of multiple regression analysis to identify factors contributing.

	to identify factors contributing to increased mean glucose level in HbA1c ≥7.0 patients.			to identify factors contributing to increased mean glucose level in non-anemic patients with HbA1c<7.0			to identify factors contributing to the increase of MAGE in non-anemic patients with HbA1c<7.0			
	Univariate analysis	Multivariate analysis		Univariate analysis	Multivariate analysis		Univariate analysis		Multivariate analysis	
	P value	odds ratio (95% CI)	P value	P value	odds ratio (95% CI)	P value	P value		odds ratio (95% CI)	P value
**Age >70 years**	0.712			0.43			0.25			
**Male**	0.66			0.445			0.74			
**BMI >25 kg/m**^**2**^	0.539			0.705			0.98			
**AST >34 IU/L**	0.961			0.127			0.23			
**ALT >43 IU/L**	0.375			0.058		N.S.	0.6			
**HOMA-IR >2.5**	0.415			1			0.09		14.716 (1.177–184.023)	0.037
**LC**	0.041	2.908 (1.031–8.204)	0.044	0.023	6.25 (1.213–32.214)	0.03	0.02		13.731 (1.334–141.326)	0.028
**HCV**	0.42			0.256			0.14			
**HBV**	0.617			0.541			0.44			
**alcohol**	0.844			0.139			0.43			
**NASH**	0.47			0.617			0.07			N.S.
**Hypertension (yes)**	0.57			0.45			0.71			
**Dyslipidemia (yes)**	0.382			0.082		N.S.	0.6			
**Hyperuricemia (yes)**	0.217			0.163			0.57			
**Treatment for diabetes (yes)**	0.61			0.5			0.57			

For abbreviations, see Table 1

### Comparison of CGM parameters between CH group and LC group in patients with HbA1c levels of < 7.0%

This subgroup consisted of 14 CH patients and 15 LC patients (Table 1). The levels of AST (*P* = 0.002) and ALT (*P* = 0.026), the rate of HCC (*P* = 0.042), APRI (*P* < 0.001), and the rate of patients on BCAA supplementation (*P* = 0.014) were significantly higher in LC group than in CH group. On the other hand, Hb levels (*P* = 0.012), platelet count (*P* = 0.006), albumin (*P* < 0.001) and HbA1c levels (*P* = 0.035) were significantly lower in LC group than in CH group. However, FPG, FIRI, HOMA-IR and the rate of glucose-lowering agents use were similar in the both groups.

**Fig 3:**
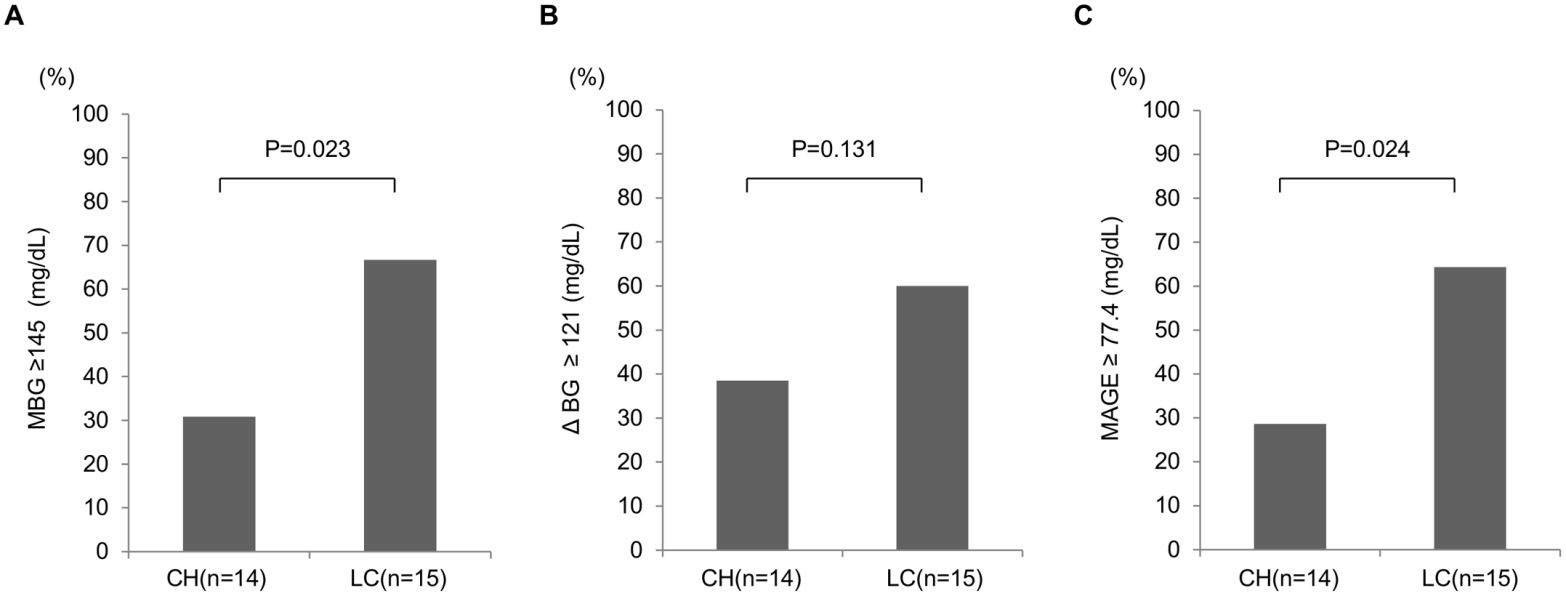
Comparison of CGM parameters between CH group and LC group among non-anemic patients with HbA1c levels of < 7.0%. (A) MBG, (B) ΔBG, and (C) MAGE. The population of patients whose values were more than or equal to the cutoff values were compared between CH patients and LC patients. MBG: mean blood glucose, ΔBG: the difference between the highest blood glucose and the lowest blood glucose, MAGE: mean amplitude of glycemic excursion, CH: chronic hepatitis, LC: liver cirrhosis.

We also compared various parameters obtained from CGM between CH group and LC group (Fig 3). ΔBG was not significantly different between CH group and LC group. On the other hand, the percentage of LC patients with MBG levels of ≥ 145 mg/dL was significantly higher than that of the CH patients (*P* = 0.023). In the same way, the percentage of LC patients with MAGE of ≥ 77.4 mg/dL was significantly higher than that of CH patients (*P* = 0.024). HbA1c levels were significantly lower in LC group than in CH group. Nevertheless, MBG and MAGE of LC group were rather higher than those of CH group.

Univariate and multivariate logistic regression analyses were performed to identify factors that contributed to the increase in MBG levels in non-anemic patients with HbA1c levels of < 7.0% (Table 2). LC was the sole factor that was independently associated with the increase of MBG levels in multivariate analysis (odds ratio = 6.250, 95% confidence interval = 1.213–32.214, *P* = 0.028). Next, we performed univariate and multivariate analyses to identify factors that contributed to the increase in MAGE in non-anemic patients with HbA1c levels of < 7.0% (Table 2). HOMA-IR (odds ratio = 14.716, 95% confidence interval = 1.177–184.023, *P* = 0.037) and LC (odds ratio = 13.731, 95% confidence interval = 1.334–141.326, *P* = 0.028) were identified as independent factors associated with the increase in MAGE.

### Postprandial hyperglycemia and nocturnal hypoglycemia

As previously mentioned, AUCgluc ≥ 140 increased significantly in proportion with worsening of hepatic functional reserve. However, there was no significant difference in AUCgluc < 70 due to differences in hepatic functional reserve. Therefore, we examined more about postprandial hyperglycemia and nocturnal hypoglycemia. Postprandial hyperglycemia was defined as maximum blood glucose of ≥ 200 mg/dL, while nocturnal hypoglycemia represented minimum blood glucose of ≤ 70 mg/dL during the night. Although many of the cases were being treated with glucose-lowering agents, postprandial hyperglycemia was noted in 92% of the patients. Nocturnal hypoglycemia was recorded in 22% of patients. Furthermore, postprandial hyperglycemia and nocturnal hypoglycemia were investigated in CLD patients with HbA1c levels of ≥ 7.0% and non-anemic CLD patients with HbA1c levels of < 7.0%. In both subgroups, the percentage of postprandial hyperglycemia or nocturnal hypoglycemia was not different between CH group and LC group (Table 3). Nocturnal hypoglycemia was seen not only in LC patients but also in CH patients. Postprandial hyperglycemia was seen at a high rate even in patients with good HbA1c levels. We could obtain these findings only by the CGM.

**Table 3:** The proportion of patients with postprandial hyperglycemia and nocturnal hypoglycemia.

		n	Postprandial hyperglycemia(%)	Nocturnal hypoglycemia(%)
**All patients**	**CH**	51	88	28
	**LC**	54	96	16
	**all**	105	92	22
**Patients with HbA1c of ≥7.0%**	**CH**	33	88	27
	**LC**	31	97	13
	**all**	64	92	20
**Non-anemic patients with HbA1c<7.0**	**CH**	14	86	36
	**LC**	15	93	33
	**all**	29	90	34

For abbreviations, see Table 1

## Discussion

The present study estimated the glycemic parameters in CLD patients with T2DM using the CGM systems. The CGM enables to evaluate short-term glycemic variability which cannot been captured by HbA1c levels, and thus, we can easily detect postprandial hyperglycemia and nocturnal hypoglycemia. Short-term glycemic variability was assessed by measuring MAGE of all readings during the CGM. Analysis of data of all cases showed no differences in various markers of T2DM, including HbA1c levels, between CH group and LC group, whereas MBG, ΔBG and AUCgluc ≥ 140 increased significantly in proportion with worsening of hepatic functional reserve. These results indicate worsening of glucose intolerance and insulin resistance with deterioration of hepatic functional reserve. High rates of nocturnal hypoglycemia and postprandial hyperglycemia were seen irrespective of HbA1c levels and liver pathology (i.e., CH or LC). Nocturnal hypoglycemia and postprandial hyperglycemia seemed to be features of CLD patients. Among patients with HbA1c levels of ≥ 7.0%, the percentage of LC patients with MBG levels of ≥ 165 mg/dL was significantly higher than that of CH patients. Among patients with HbA1c levels of < 7.0%, the percentage of LC patients with MBG levels of ≥ 145 mg/dL was significantly higher than that of CH patients. Likewise, the percentage of LC patients with MAGE of ≥ 77.4 mg/dL was significantly higher than that of CH patients. Thus, regardless of the HbA1c levels, glycemic parameters derived from CGM were much worse in LC group than in CH group. In summary, CGM analysis of CLD patients with T2DM demonstrated aggravation of glycemic variability (as reflected by MAGE, MBG, ΔBG etc.) with deterioration of liver functional reserve.

Insulin resistance and hyperinsulinemia are metabolic features of patients with CLD [21]. Hyperinsulinemia in these patients is caused by either hepatic cell damage or portal-systemic shunting because blood glucose is delivered to the liver through the portal vein. The rate at which insulin is metabolized in the liver is reduced in patients with CLD. Moreover, despite peripheral hyperinsulinemia, insulin levels in the portal veins are low in CLD patients with portal systemic shunting [22–24]. The frequent blood measurements have not able to reflect the various glycemic kinetics in CLD patients. The CGM systems enable to evaluate such glycemic variety in CLD patients.

Both HbA1c and GA are less reliable markers in CLD patients with T2DM. We divided the patients into two groups; those with HbA1c levels of ≥ 7.0% and the other with HbA1c levels of < 7.0%. In the former group, various markers of T2DM, such as HbA1c, FPG, FIRI, and HOMA-IR were not significantly different between CH and LC groups. Moreover, the proportion of patients using glucose-lowering agents was significantly higher in CH group than in LC group. Nevertheless, the percentage of patients with high MBG levels was significantly higher in LC group than in CH group. Since similar HbA1c levels can be generated by different glucose profiles because HbA1c is an integrated measure of overall glucose exposure [12], these results suggest that among patients with comparable HbA1c levels, the actual average blood glucose or blood glucose fluctuations in LC patients with poor liver reserve were higher than those of CH patients. Multivariate analysis identified LC to be significantly associated with increased prevalence of high MBG levels. On the other hand, in CLD patients with HbA1c levels of < 7.0%, the percentage of patients with high MBG levels or high MAGE of LC group was significantly higher than that of CH group, despite their HbA1c levels were significantly lower than those of CH group. Multivariate analysis identified LC as significantly associated with increased prevalence of high MBG levels. Moreover, by multivariate analysis, LC and the presence of insulin resistance as significantly associated with the increased prevalence of high MAGE. These results suggest that the existence of CLD patients with satisfactory glucose control markers develop large blood glucose fluctuations or have high MBG levels. Furthermore, LC and insulin resistance were significantly associated with these phenomena. In the case without anemia with normal HbA1c levels and seemingly good glucose control, we have slipped the patients in need of treatment intervention. We could not find the abnormality of such hidden blood glucose fluctuations without CGM. The results based on CGM help for the management of T2DM in CLD patients.

Although many of the cases were being treated for T2DM, postprandial hyperglycemia was seen in 90% of non-anemic patients with HbA1c levels of < 7.0%. Nocturnal hypoglycemia was seen in 20% of patients with HbA1c levels of ≥ 7.0%, and in 34% of non-anemic patients with HbA1c levels of < 7.0%. To identify such cases, the existing glucose metabolism markers, such as HbA1c, were not useful. Because nocturnal hypoglycemia may remain asymptomatic, it could not be recognized without CGM.

Our study has several limitations. First, we did not evaluate CLD patients without T2DM, because medical insurance do not cover for CGM in non-diabetic patients. It is possible that postprandial hyperglycemia and nocturnal hypoglycemia exist in patients with CLD who do not meet the diagnostic criteria for T2DM. We expect to be allowed to use of CGM examination in the future in CLD patients without T2DM. Second, the study did not evaluate the role of each etiology of CLD due to the small number of patients. Finally, we could not evaluate the glycemic parameters of patients without any liver disease as control group because only patients with some liver disease visit in our department.

## Conclusion

In conclusion, this present study showed that in CLD patients with T2DM, the decrease in liver functional reserve is associated with worsening of parameters of glycemic variability including MAGE, MBG, ΔBG, etc. The CGM systems were useful in finding hidden abnormalities of blood glucose fluctuations in CLD patients with T2DM, especially in non-anemic CLD patients with HbA1c levels of < 7.0%. Furthermore, the CGM systems were useful in detecting asymptomatic nocturnal hypoglycemia in CLD patients. Information obtained from the CGM should be used to customize treatment to reduce episodes of nocturnal hypoglycemia by which to maintain the liver functional reserve.
